# Osteopathic Manipulative Treatment to Address Post-Cholecystectomy Diarrhea in the Peruvian Amazon: A Case Series

**DOI:** 10.51894/001c.165593

**Published:** 2026-07-31

**Authors:** Derek Esty, Olivia Dunmore, Lauren Gillette, Sophie Bair, Brad Draher, Grace Larsen, Cydney Pittenger, Kisa Seerveld, Travis Gordon

**Affiliations:** 1 College of Osteopathic Medicine Michigan State University

## 79

### Introduction

Cholecystectomy is one of the most common abdominal surgeries, with 750,000-1,000,000 performed annually in the United States. Post-cholecystectomy diarrhea (PCD) is a common sequela of cholecystectomy and is characterized by chronic, loose stools three or more times daily that begin weeks to months after cholecystectomy. PCD occurs in approximately 10–15% of patients and can significantly reduce quality of life. Treatment for PCD includes bile acid resins, which present compliance challenges related to common side effects. Osteopathic manipulative treatment (OMT) has demonstrated significant benefit in gastrointestinal and postsurgical conditions, but its role in PCD remains unclear.

### Case Description

Six patients presenting to an annual MSUCOM outreach at the Clinica San Martin de Porres in Iquitos, Peru, were evaluated and treated. Patient ages ranged from 36-73 years. All patients reported new onset diarrhea within weeks after cholecystectomy, persisting until time of evaluation. Chronicity of symptoms varied between 3 and 20 years. Patients reported multiple loose stools daily, abdominal pain/cramping, bloating, nausea, and/or food intolerance. Stool consistency was assessed using the Bristol Stool Form Scale (BSFS), and depression and anxiety symptoms were screened with PHQ-9 and GAD-7. The six patients were treated with indirect myofascial release technique directed to the cholecystectomy surgical scar and adjacent abdominal tissues.

Clinical follow-up was done by phone 2-3 weeks post-treatment, revealing significant improvement in PCD. The average BSFS score decreased from 5.5 to 4.2, indicating an improvement from loose to formed stool. Food intolerances improved or resolved in five of six cases. The mean PHQ-9 scores improved from 11.33 to 4.67, all six subjects showing improvement. The mean GAD-7 scores improved from 11.77 to 5.00, five of six subjects showing improvement.

### Discussion

This case series demonstrates significant improvement in biomedical and psychosocial outcomes after a single OMT session, suggesting OMT may be a viable, low-cost, non-pharmacologic treatment for PCD. This is particularly encouraging given the relatively high incidence of PCD, its difficulty to treat, and significant reduction in quality of life experienced by patients. Prospective trials with larger sample sizes would be necessary to verify efficacy of OMT in treating PCD.

